# Enhanced education and support needs in rheumatoid arthritis associated interstitial lung disease (RA-ILD) – patient experiences from a multicentre UK survey

**DOI:** 10.1007/s00296-025-05988-z

**Published:** 2025-09-24

**Authors:** Shirish Dubey, Abhinav Peddasomayajulu, Anupama Nandagudi, WinWin Maw, Damodar Makkuni, Siwalik Banerjee, Gouri M. Koduri

**Affiliations:** 1 Dept of Rheumatology, Oxford University Hospitals NHS FT, Windmill Road, Oxford, OX3 7HE UK; 2 Nuffield Department of Orthopaedics, Rheumatology and Musculoskeletal Sciences, University of Oxford, Windmill Road, Oxford, OX3 7LD UK; 3 Southend University Hospital, Prittlewell chase, Southend on sea, SS0 0RY UK; 4 Basildon University Hospital, Nether Mayne, Basildon, SS16 5NL UK; 5 Anglia Ruskin University, Bishop Hall Ln, Chelmsford, CM1 2SQ UK; 6 Broomfield Hospital, Court Rd, Broomfield, Chelmsford, CM1 7ET UK; 7 James Paget University Hospital, Lowestoft Rd, Gorleston-on-Sea, Great Yarmouth, NR31 6LA UK; 8 Rheumatology department, University Hospital Coventry and Warwickshire NHS Trust, Coventry, CV2 2DX UK

**Keywords:** RA-ILD, CTD-ILD, SARD-ILD, Interstitial lung disease, Surveys and questionnaires, Patient education, Outcomes, Patient perspectives, Patient experience, Outcomes, Rheumatoid Arthritis

## Abstract

**Objectives:**

Interstitial lung disease (ILD), one of the complications of rheumatoid arthritis (RA) has significant impact on morbidity and mortality. Very little work has been done on patient perceptions, experiences and their needs in RA-ILD. This study aimed to fill that gap in order to better understand and optimise care pathways.

**Methods:**

There are no validated questionnaires, so we piloted and developed one based on Commissioning for Quality in RA Reported Experience Measure (CQRA-PREM). This study was conducted at 6 sites following formal ethics approval. Patients with RA-ILD were identified from routine clinics and databases.

**Results:**

We included 64 completed valid responses in the final analysis. Median age of the cohort was 75 years; duration of RA was 7 years. Only 13 (20%) participants received detailed information on ILD. Majority reported negative experiences regarding their involvement in care (*n* = 40, 64%) and needed help from family members or carers (*n* = 35, 60%). Half were attending respiratory clinics regularly (*n* = 34, 53%) or having regular PFTs (*n* = 29, 45%). Only 11 (17%) were able to do moderate exercise or higher. Participants desired more information on ILD, frequent appointments with specialists, earlier referral to specialist centre, and improved communication between specialists.

**Conclusions:**

This study explores patient perspectives in RA-ILD across 6 different UK socioeconomic areas. There are substantial educational needs, disability, and notable gaps in service provisions. Enhanced patient support is needed, and this necessitates more effective integration and utilisation of the multidisciplinary team, including specialist nurses, psychologists, pharmacists, and other allied health professionals.

**Supplementary Information:**

The online version contains supplementary material available at 10.1007/s00296-025-05988-z.

## Introduction

Rheumatoid Arthritis (RA) is a chronic inflammatory disease with a prevalence around 1% and a peak onset of disease is between 45 years and 64 years of age. More than 400,000 people in the United Kingdom are affected [1]. The economic burden of RA is high due to joint damage, deformity, work disability and extra-articular complications [2]. Although joint disease is the main presentation, extra-articular manifestations of RA are common affecting up to 40% of patients that contribute to the substantial morbidity and excess mortality [3]. Furthermore, life span of RA patients is shortened by approximately 10 years with cardiovascular disease being the leading cause of death, followed by interstitial lung disease (ILD) as the second most common cause [3, 4].

RA can affect the lung parenchyma, and this typically occurs in the first few years of diagnosis. ILD can also precede the development of joint problems in about 10% of patients [5–10]. Prevalence rates of RA - ILD vary based on the diagnostic technique and reported from 19 to 70% [10], although more recent data suggest lower prevalence rates [11]. Patients with RA-ILD have a heterogeneous clinical presentation with an unpredictable disease course. The median survival for RA patients diagnosed with ILD is 2.5 to 7 years, particularly worse in Usual Interstitial Pneumonia (UIP) pattern although the range is wide, some following a slowly declining trajectory over many years whilst some others may have a rapidly progressive course [6, 12, 13]. A recent meta-analysis confirmed the prognostic significance of UIP pattern [14]. A review published in 2021 lamented the lack of consensus on risk and prognostic factors [15]; however more recent work has identified that the most common risk factors for development of ILD are older age, males, smokers, strongly positive antibodies and to a lesser extent RA disease activity [16], further work is ongoing.

Significant gaps persist in the evidence base regarding optimal screening methodologies, monitoring strategies, physical and mental health needs and therapeutic considerations for RA-ILD care. Patients with ILD are confronted with both the physical limitations imposed by the disease and the psychological impact of an ultimately fatal condition with uncertain rate of progression. In addition, RA can also have significant impact on their QoL. A review [17] highlighted studies that have attempted to measure or improve QoL in Idiopathic Pulmonary Fibrosis (using patient-reported outcome measures and interventions) and those that have identified unmet patient needs (such as emotional support and information resources). In Idiopathic Pulmonary Fibrosis (IPF), there are a few studies now that have assessed the individual psychological impact of this diagnosis and patients’ experiences of living with the disease [19–23]. In RA-ILD, there remains a paucity of literature on patient perception of the disease, their understanding and needs. Patient-centred care is gaining more attention in the last decade and has become a key part of care organization in chronic diseases. In COPD care, self-management strategies have been shown to be of benefit in health-related quality of life (HRQoL) and dyspnoea and also demonstrated a reduction in hospitalizations [23]. Similarly, self-management strategies have been implemented for IPF and these have shown improvements in some domains such as 6-min walk distance, St George’s Respiratory Questionnaire and the Medical Research Council Dyspnoea scale [24]. In RA -ILD care, no studies have investigated or explored the impact of self-management strategies or patients’ coping mechanisms and there are very limited studies on patient experiences and perceptions (mainly in Connective Tissue Disorder (CTD) related ILD). This highlights the need for further research on self- management needs and interventions in RA-ILD. Hence, we initiated this study in order to gain a deeper understanding of the impact of RA-ILD on patients’ lives, and to identify their specific needs. The aim of this study was to explore patients’ experiences of living with RA associated ILD, evaluate current care provision, and examine the physical, medical and psychosocial impact of the disease. Additionally, we sought patients’ perspectives on priorities for future service development.

## Patients and methods

This was a non-web-based questionnaire study with open ended questions to elicit detailed patient responses; therefore, it was classed as semi-qualitative. Patient-Reported Experience Measures (PREMs) are either generic or disease specific. As there are no validated questionnaires (for RA-ILD or CTD-ILD) currently, we developed a questionnaire, based on Commissioning for Quality in RA Reported Experience Measure (CQRA-PREM) [25] principles to address the specific aims of this research project.

Key themes included patient knowledge of ILD, perception of the condition, experiences with medication and overall management. Two patients from National Rheumatoid Arthritis Society (NRAS) group were involved in designing the questionnaire. The first version of the questionnaire was developed on 28th September 2022, this was exploratory in nature and was piloted with ten patients to ensure clarity and relevance. They found questions easy to understand and they thought questions were relevant. The average time to fill in the questionnaire was around 9 min. Further adjustments were made based on the feedback and final version was submitted alongside responses to the ethics committee on 5th Jan 2023 (this is available as supplementary material). Favourable ethical approval was received (IRAS 319483, REC reference number is 22/EE/0303), dated 17th Feb 2023, from Health Research Authority and Health and Care Wales. Patients were approached face to face when attending rheumatology department for any reason. Those who expressed interest were subsequently contacted by the research team. A second recruitment pathway involved identifying potential participants from the database, after which study information and consent forms were posted to them. Patients who agreed to participate returned the signed consent forms and completed the questionnaire either during a hospital visit, by email, or via post. All study participants provided written consent. The study was performed in keeping with the principles of good clinical practice in research as per the Helsinki agreement. The study was conducted between February 2023 and June 2024.

Consecutive patients from outpatient clinics and day unit were recruited from NHS secondary care rheumatology centres - Southend, Oxford, Basildon, Broomfield, Great Yarmouth, and Coventry to represent demographic, socioeconomic and geographic diversity. All these centres have ILD-MDT (Multidisciplinary team meetings) and most have combined clinics with respiratory and rheumatology clinicians. Only anonymised data were collected from the sites; no participant identifiable information were included in the central dataset. Only paper-based questionnaires were utilised, and no financial incentives were provided to patients. Each site kept their own log with patient’s hospital number to ensure no duplication.

Eligible patients, who met the criteria of 2010 ACR/EULAR for RA and HRCT proven diagnosis of ILD were included in the study. The demographic data included age, gender, body mass index (BMI), education, smoking and employment status.

*Questionnaire*: We organized survey questions according to the phases in the care continuum, including information received about ILD (5 questions), co-ordination and communication (4 questions), impact of ILD on patient’s daily life (3 questions), extent of patientinvolvement in care and treatment decisions (3 questions), and recommendations for future care improvements (qualitative data). Data regarding pulmonary rehabilitation and other adjunctive treatment were also collected. The questionnaire was administered by the research team including doctors and nurses. Patients who had expressed willingness to participate in this were sent 2 reminders if their responses were not received in agreed time frames.

*Data analysis*: For the descriptive statistics, MS Excel was used to describe the socio-demographic characteristics of the sample, disease characteristics, treatment, and outcomes. Patient quotes were analysed as qualitative data. We utilised student’s T test to test differences between means and analysis of variance (ANOVA) to assess whether there was significant variation in responses.

## Results

Following verbal and written consent, questionnaires were provided to 121 patients. Questionnaires were posted out to patients following identification from clinics.

Amongst the 6 sites that participated, there was significant attrition with several questionnaires not returned and 5 additional responses were deleted because of minimal data. Overall, 64 completed valid responses were received and analysed.

The demographics of the participants are described in detail in Table 1. As expected, females were higher at 56% (*n* = 36) than males. Median age of the cohort was 75 years (IQR 13.25). Median duration of RA since diagnosis was 7 years (IQR 0), the vast majority of this cohort (*n* = 56, 89%) had well established RA with very few patients (*n* = 7, 11%) in the ‘early’ category (less than 5 years of diagnosis). Majority of participants (*n* = 55, 86%) had ILD duration of less than 5 years with 7 (11%) having ILD for more than 5 years. Education status of the participants was: None for 14 (22%), GCSE 12 (19%), A level 18 (28%), University and postgraduate education 12 (19%), Not answered 8 (13%).

**Table 1:** Demographics and clinical characteristics

	*N*, %
Gender	
Male	28 (44%)
Female	36 (56%)
Age – median (years)	75
40–59	5 (8%)
60–79	41 (64%)
80 or more	18 (28%)
Smoking history	
Current/ex-smokers	38 (60%)
Never	26 (40%)
RA duration*	
< 5 years	7 (11%)
5–10 years	27 (42%)
> 10 years	29 (45%)
ILD duration*	
< 3 years	28 (45%)
3-5 3–5 years	27 (42%)
> 5 years	7 (11%)
Treatment changes*	
No change in DMARDs	29 (45%)
DMARDs reduced/stopped	10 (16%)
Rituximab	3 (5%)
MMF	1 (1.5%)
Nintedanib	8 (12.5%)
Abatacept	1 (1.5%)
Changes in ILD*	
Improved	13 (20%)
Worsened	11 (17%)
No change	31 (48%)

*Missing data for these fields, so total numbers vary

(RA duration – 1 missing, ILD duration – 2 missing, treatment changes – 4, changes in ILD − 9)

### Information on ILD

Only13/59 (22%) participants said they received detailed information on ILD, 19 (32%) received no information and 23 (39%) received minimal information. 49 (83%) stated that they did not receive any information on website or patient groups and 14 (24%) received some information. We analysed whether gender, age group, educational attainment or attending respiratory clinics regularly impacted this but did not find any significant differences.

### Treatment

Since the diagnosis of ILD, 55/64 patients reported changes to their treatment with 3 starting Rituximab and 1 Abatacept. Nintedanib was started for 8 (12.5%) patients (this is currently available in the NHS for progressive pulmonary fibrosis), whilst 1 commenced on Mycophenolate mofetil and 4 received corticosteroids. DMARD (disease modifying anti-rheumatic drug) doses were increased for 3 patients and in 10 (16%) participants DMARDs were either reduced or stopped. Other adjunctive treatments were Carbocisteine (2) Inhalers (7), PPI (1), Oxygen (2, long term oxygen therapy), Apixaban (2) and 1 received prophylactic antibiotics. Following the treatment change, 13/55 (24%) reported some improvement and 42/55 (76%) did not notice any change or experienced worsening of ILD symptoms. Only 10/64 (16%) were referred for chest physiotherapy.

*Involvement with care and decision making*: Substantial proportion of participants had negative views (64%) on this. 24 (38%) participants said that they were not involved at all with their ILD management or decisions about their care and 16 (25%) felt they were involved just a little whilst only 14 (22%) were very much involved. Eight (13%) felt somewhat involved. Overall, only 22 (35%) participants felt part of shared decision making. Further analysis of subgroups did not demonstrate any meaningful differences.

*Follow up*: Twenty-three (36%) participants attended Respiratory clinic at 3–6 months, 11 (17%) annually, 5 (8%) only once and 18 (28%) infrequently.

Pulmonary function tests (PFTs): There was a lot of heterogeneity with 9 (14%) participants having 6 monthly tests, 20 (31%) being tested annually, 25 (39%) at random and 5 (8%) only once.

*Impact on their quality of life (QoL) and physical activities*: ILD had significant impact on QoL and 33 (52%) reported that ILD had as much impact on their QoL as joint problems, and 37 (57%) needed help from their family and carers. Help could be in the form of support for shopping, physical, emotional and financial needs. Fifteen (23%) participants received information on self-management and 28 (44%) were provided with helpline number to discuss about their condition and treatment.

*How did they stay physically active*: Majority were able to walk and take light exercises (40 out of 64, 62.5%). Thirteen (20%) participants could not undertake any activities and were housebound. Eight (12.5%) were able to undertake moderate exercises including Cardio strengthening, yoga, Pilates, circuit training and swimming. Three (5%) were still in full time employment. Seventeen (27%) were able to undertake light activities such as housework, gardening, growing vegetables. Two patients were on long term oxygen therapy (LTOT). Thirty-eight (59%) participants were able to undertake small walks, including dog walking and in the garden.

Overall, detailed subgroup analysis stratified by gender, educational attainment, recent ILD diagnosis and regular attendance at respiratory clinics did not reveal any statistically or clinically meaningful differences.

### Qualitative data

**Fig. 1:**
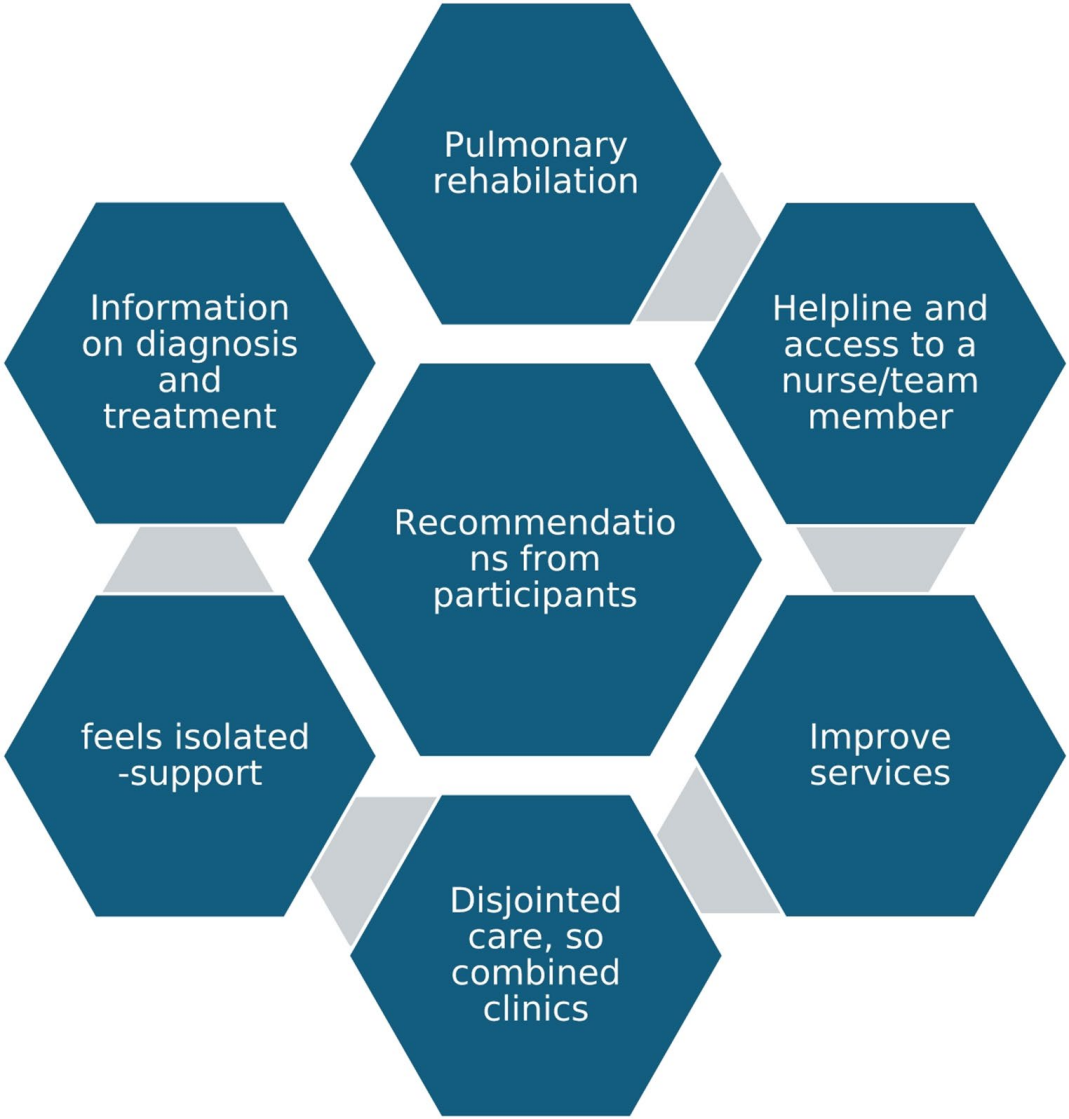
Recommendations from participants

The qualitative data was derived from responses to open ended questions and free text comments. Recurring responses were grouped into key themes. Some of the comments reflected the dissatisfaction with changing nature of professional consultations (non-face to face appointments), highlighting the perceived decline in the quality of professional interactions. Many participants expressed their need for more information on ILD, frequent appointments with a specialist, early referral to specialist centre and better communication between specialists - see Fig. 1. Interestingly, 18 (31%) participants stated that they did not have enough knowledge to make recommendations, this was numerically more likely amongst people with lowest educational attainment (10 out of 14, 71%). Analysis of variance, however, did not reveal significant differences (*p* = 0.40).

**Fig. 2:**
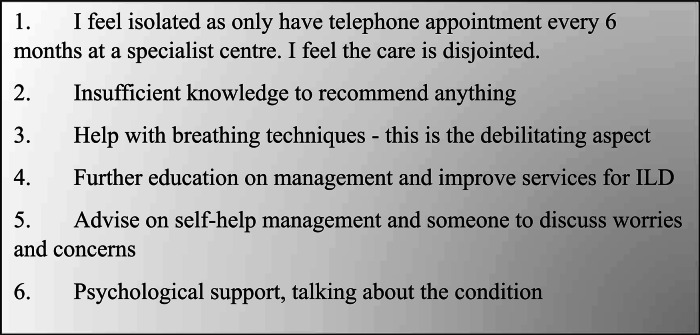
Representative quotes from patients

Figure 2 includes some quotes that represent the various themes from the responses.

## Discussion

Our multicentre study identified key issues: lack of understanding of ILD amongst participants, variability in care received and significant gaps in care provisions. Majority of participants reported feeling uninvolved in decisions about their care, a damning reflection of the current practice.

Although the literature on RA-ILD is limited, some findings from older studies (CTD-ILD, IPF) from several years ago are consistent with our results. Participants expressed a clear need for enhanced support, better communication between specialists and greater involvement of the multidisciplinary team, including specialist nurses, psychologists, pharmacists, and other allied health professionals. About a third of our participants did not feel that they could make recommendations for care, noticeably higher proportion of people from less educated backgrounds responded in this manner.

We performed a comprehensive search using several databases: Knowledge and Library Hub, OVID, The Patient Experience Library, CINAHL, Cochrane, BNI, AMED, PsycInfo, Google/Google Scholar. This confirmed the absence of major studies evaluating patient perspectives in RA-ILD. Only one recent, a single centre mixed methods study examined RA PREM in ILD population, and it identified twenty-four statements representing the eight domains of the RA-PREM. It met face/content validity criteria, however longitudinal validation is needed for its reliability and the findings have not yet undergone peer review [26]. Additionally, we identified one relevant conference abstract from 2012, which reported patient perspectives based on a focus group of seven patients with RA-ILD in Toronto (Canada) [27]. Mittoo et al. [28] conducted a mixed method study in CTD-ILD patients with 45 participants: IIM-ILD (Idiopathic Inflammatory Myopathy-ILD, *n* = 11), RA-ILD (*n* = 13), SSc-ILD (Systemic sclerosis–ILD, *n* = 17), and various other CTD diagnoses (*n* = 4). A lack of sufficient disease related information was highlighted, with participants expressing a desire for detailed information on their lung status, and access to support groups. In SSc-ILD, two studies have explored patient perspectives. One of these focused on shared decision making through discrete clinical experiments and patients were willing to accept certain side effects in exchange for improvement in respiratory symptoms [29]. The other study reported lack of education and constant worries about the long-term disease consequences [30]. Our findings are consistent with these studies, reinforcing that limited information on ILD and insufficient access to support groups remain key unmet needs.

Very few studies have examined the impact of RA-ILD on health-related quality of life (HRQoL). One study compared HRQOL in RA ILD and IPF using SF 36, demonstrated that the physical components were worse in RA-ILD than IPF [31]. Another study from China found significantly worse HRQoL in RA -ILD compared to RA without ILD [32]. In our study, 52% reported a significant impact on QoL, with ILD affecting quality of life as much as articular disease. Most were only able to walk and undertake light exercise, possibly a consequence of combined of joint and lung involvement, there might be other factors, which were not explored in our study. Disability and quality of life were also prominent in Mittoo et al. CTD -ILD study [28]. These findings highlight the need for early intervention in these patients with exercise-based interventions to reduce the disability across this group of patients.

Our study has highlighted educational gaps, and many patients felt insufficiently informed about RA -ILD and unable to contribute meaningfully to decisions about their care. Shared decision-making is a core NHS principle [33], requiring that patients have the knowledge, skills, and confidence to manage their condition.

A web-based survey investigating 258 RA patients in USA reported that only 26% of patients were satisfied with their RA care despite majority of these patients using biologics [34]. High rates of co-morbidities, mental health concerns (44%) and high disease activity were reported in this study (33% of patients were in high disease activity state as per RAID (Rheumatoid Arthritis Impact of Disease) scoring). Another qualitative study of early RA concluded that genuine person-centred care is important not only in early stages but should be implemented throughout the healthcare system [35]. In our study, patient engagement was similarly poor, many patients expressed negative experiences and with only a minority feeling actively involved in their care. Although not captured within this study, it is evident that education for healthcare professionals unfamiliar with RA -ILD, could improve patient experiences.

Currently, good quality information exists for RA and for IPF, but none exist specifically for RA -ILD or other CTD -ILDs despite the considerable impact of these conditions on morbidity and mortality. Educational needs for RA subjects are often greater than in other chronic conditions [36]. These needs of patients need to be taken seriously and addressed through information leaflets focused on RA-ILD, face to face Consultations, nurse led education sessions and online resources including videos for patients.

Participants expressed disappointment with poor communication from the healthcare providers, delayed referral to specialist centres and lack of contact point or helpline/adviceline to discuss their disease progression, symptom management, concerns and anxieties relating to breathing difficulties. Similar findings were reported in other limited studies and highlights the urgent need to develop and optimise support resources in CTD-ILD [28]. Education on ILD needs a lot more focus with both teams (respiratory and rheumatology) playing an active role, particularly in discussion around pharmacotherapeutic options.

In our study participants have recommended frequent appointments with a specialist, early referral to specialist centre and better communication between specialists. Service models vary across the UK from joint clinics with both specialists present, to co-located services or no formal collaboration. Multi-disciplinary team meetings have become the norm since the NICE recommendations for IPF [37]. The best model for management of these patients remains to be established and needs formal evaluation.

RA-ILD is unique among CTD-ILDs in involving two organ systems, with respiratory complications often being the dominant cause of morbidity and mortality. It is important to recognise the different patterns of lung involvement in RA-ILD, such as Usual Interstitial Pneumonia (UIP; commonest), organising pneumonia (OP) and Non-Specific Interstitial Pneumonia (NSIP). Disease progression in RA-ILD typically differs from that seen in other CTD-ILDs. Patients with RA should be screened for ILD at every clinical encounter [14].

Methotrexate is the commonest DMARD used in RA, it was often avoided but has now been shown to reduce the incidence of ILD [38]. The treatment of ILD has evolved over the last decade with discovery of antifibrotics and specific therapies for SSc-ILD but robust clinical trials in RA-ILD are lacking. Given the differing patterns of ILD, a multicentre umbrella or platform trial might be the optimal strategy for generating the evidence base needed for optimising patient care.

In our study, the proportion of patients moving on to advanced therapies such as biologics and anti-fibrotics seems quite small, also several patients were not under respiratory care despite proven ILD which is concerning. The underlying reasons for this are not entirely clear, but it is likely that the lack of appropriate monitoring and follow up was contributory. It is estimated that 40% of RA-ILD patients are at risk of developing progressive pulmonary fibrosis (PPF) [39], so early intervention and regular monitoring becomes paramount. In addition, pulmonary rehabilitation, psychological support, and advanced care planning/palliative care should be incorporated into routine care pathways. The SMILE RA programme developed by National Rheumatoid Arthritis Society (NRAS) for general RA education is a step forward, similar modules are needed for patients with RA-ILD [40].

### Limitations

This was a questionnaire-based cross sectional study, and these kinds of studies have intrinsic limitations due to the study design. The numbers in this study are small, and lack of outcome data can provide a biased viewpoint. As no validated questionnaire existed for assessing patient experience of RA–ILD, we developed items based on the CQRA-PREM. While content, construct and face validity were addressed through literature grounding, patient involvement and piloting and revising, however, reliability (e.g., test–retest, internal consistency) was not assessed. Due to low recruitment numbers, we were unable to perform detailed subgroup analyses and therefore limited our approach to descriptive analysis. This methodological limitation may influence generalisability. However, geographic diversity of responders supports the broader relevance of our results despite a 53% response rate. It is our intention to develop and validate a dedicated RA -ILD patient experience measure in a large prospective study.

## Conclusions

This is the first study to provide a detailed assessment of patient perspectives in RA-ILD, conducted across six UK sites with diverse geographic and socio-economic backgrounds. Our findings reveal substantial unmet educational needs, considerable disability and significant deficiencies in current care provision. Patients expressed a strong desire for improved education, greater support and more collaborative patient centred approaches to care. We recommend the implementation of comprehensive education initiatives, standardised care pathways, greater patient involvement in decision-making, and overall improvements in the quality of care for individuals with ILD.

## Supplementary Information

Below is the link to the electronic supplementary material.

Supplementary Material 1

## Data Availability

Further data can be made available at request, please contact the corresponding author.
