# Supplementary material for: Enhanced education and support needs in rheumatoid arthritis associated interstitial lung disease (RA-ILD) – patient experiences from a multicentre UK survey
Source: Rheumatol Int. 2025 Sep 24;45(10):237. doi: 10.1007/s00296-025-05988-z (PMC12460471; doi:10.1007/s00296-025-05988-z)
Supplement: Supplementary file 1 — Supplementary Material 1 [file 296_2025_5988_MOESM1_ESM.pdf]

Questionnaire **Recruiting site ……….**
Principal Researcher: GMK
Version 1.0. 05/01/2023

Prospective non- interventional exploratory study to assess patients' experiences and coping with Rheumatoid Arthritis – Interstitial Lung Disease (ILD) and their recommendations

Questionnaire

Demographics

1. Age …………………. ………………………………………………….

2. Sex Male ……………… Female……………………………………….

3. Marriage Status: Married/Divorced/ Widowed/ Separated/ Cohabiting/ Single

4. Height…………. …………………. Weight …………………

5. Smoking history
   Current 1 - 5 years ……..… 5 -10 years………… > 10 years …………

   Ex -Smoker gave up < 12 months …… 1- 5 years……… > 5years………
   Never…………….

6. Your highest level of education?

GCSE/A level or equivalent ………… Degree or equivalent…………………
Postgraduate qualification ……………………………………….

7. Employment status - Full time/part time/retired//manual/semi-manual/sedentary office Job/Not employed

8. How long have you been suffering from Rheumatoid Arthritis?
   …………………….

9. Tell us when were you diagnosed with ILD? Tick one of the options
   <1 year…………., 1-3 years……………., <5 years…………………

Information and treatment

1. How much information was provided on ILD and management?

   No information/Minimal Information/Detailed Information/Too much information/Not sure

2. a. What treatment are you on Rheumatoid Arthritis?
   DMARDs/ Steroids/Ant -TNF/Rituximab/Abatacept /JAKI/TCZ

   b. What treatments changes were made since the diagnosis of ILD?

   …………………………………………………………………….

3. Are you on Oxygen ………… Prophylactic antibiotics …………Any other medications for ILD …………?

4. The treatment for ILD has

   Improved…………. worsened…………… no change………………

5. Were you given information on websites on ILD, patient organizations or groups?

   Yes………………………………………., No …………………………………

Coordination and communication

6. How often do you attend Respiratory clinic?
   Only once……. …3 months……. 6 months………. 12 months…………
   infrequent……………

7. How often do you have Lung Function tests?

   6 months…………. annual…………. random……………………

8. To what extent were you are involved in your ILD care or about the decisions about your care?

   Not at all…………Just a little ……………. Somewhat……………Very much………….

9. Have you received advice from chest physiotherapy?
Yes………………………. No………………………….

Impact on quality of life

10. Does ILD impact as much as Rheumatoid Arthritis on your Quality of life?
Yes …… …………………… No……………………..………….

11. Do you need help from family members/carers? (e.g., Shopping, Physical, emotional, financial)
Yes ………………………. No……………………………………

12. How do you stay physically active?
……………………………………………………………………………………..

<u>Education and self-care</u>

13. Were you given information on self-management program?
Yes………………………. No……………………

14. Were you given helpline number or able to approach a member of the team to discuss any worries about your condition and treatment?
Yes………………………… No…………………………………

15. What would you recommend in order to improve the disease management or services?
……………………………………………………………………………………
……………………………………………………………………………………
